# Systemic arterial hypertension as a risk factor for the severe form of covid-19: scoping review

**DOI:** 10.11606/s1518-8787.2022056004311

**Published:** 2022-03-29

**Authors:** Ana Cristina Ribeiro, Sílvia Carla da Silva André Uehara

**Affiliations:** I Universidade Federal de São Carlos Departamento de Enfermagem São Carlos SP Brasil Universidade Federal de São Carlos. Departamento de Enfermagem. São Carlos, SP, Brasil

**Keywords:** COVID-19, complications, Risk Factors, Hypertension, Review

## Abstract

**OBJECTIVE:**

Identify whether systemic arterial hypertension is a risk factor for the severe form of covid-19.

**METHODS:**

This is a scoping review, searches were performed on the Lilacs, PubMed, SciELO and Web of Science databases.

**RESULTS:**

Thirteen articles were selected. The studies presented systemic arterial hypertension as the most common chronic disease in subjects diagnosed with covid-19. Hypertensive subjects were older, and men were more likely to develop severe covid-19. Hypertensive subjects without antihypertensive treatment were associated with a higher risk of mortality.

**CONCLUSIONS:**

subjects with chronic diseases tend to have a different clinical profile. Blood pressure should be controlled in hypertensive subjects that should be continuously monitored during the covid-19 infection.

## INTRODUCTION

Systemic arterial hypertension (SAH) affects more than 30% of the adult population, or more than one billion people, and is the leading cause of premature death worldwide^1,2^. The burden of disease disproportionately affects low- and middle-income countries, where two-thirds of cases are found. This is largely due to the increase of risk factors in these populations in recent decades. Approximately half of people living with hypertension is unaware of their condition, putting them at risk for preventable medical complications and death^2^.

Hypertension is considered a chronic non-communicable disease (NCD). It is a multifactorial condition that depends on genetic and epigenetic, environmental and social factors. Its cut-off point is defined by systolic blood pressure (SBP) ≥ 140, and diastolic blood pressure (DBP) ≥ 90 mm Hg^3^. According to the Brazilian Guidelines on Hypertension - 2020, some of the main risk factors for developing SAH include genetics, aging, gender, ethnicity, overweight and/or obesity, high sodium intake, sedentary lifestyle, alcohol intake, as well as socioeconomic factors, including lower education, inadequate housing conditions, and low family income^3^.

Recent studies point out that the existence of comorbidities, such as systemic arterial hypertension, in subjects with covid-19 could lead to unfavorable outcomes, including an increased risk of death^4^. In this scenario, studies have identified that hypertension is the most common chronic disease in subjects infected with the new coronaviruses, which may be justified by the high global prevalence^7^.

Subjects with systemic arterial hypertension experience endothelial dysfunction, which appears as an imbalance between vasodilator and vasoconstrictor substances that directly affects vascular function. This is considered to be a major characteristic of the vascular bed in hypertensive subjects. Associated with the aging process, hypertension results in progressive stiffening and loss of compliance of the great arteries, and is crucial in the pathogenesis of cardiovascular complications related to the covid-19^11,12^.

It is noteworthy that since SAH is often asymptomatic, subject may evolve to structural and/or functional changes in target organs such as heart, brain, kidneys, and vessels. This would increase susceptibility to the SARS-CoV-2, increasing the risk of unfavorable outcomes in people with covid-19^3,13^.

Data on the impact of known and newly diagnosed hypertension in subjects with covid-19 are still very limited. One study, however, showed that insufficient control of blood pressure (BP) was independently associated with adverse outcomes in covid-19 and hypertensive subjects^14^.

Another study reported that stage I hypertension was present in 37% of subjects hospitalized for covid-19, while the prevalence of stage II and III hypertension was significantly higher (61% and 70%, respectively). Unfavorable outcomes (mortality, septic shock, respiratory failure, acute respiratory distress syndrome [ARDS], and intensive care unit admission) gradually increased with elevated blood pressure^15^.

It is also noteworthy that most hypertensive subjects require pharmacological treatment, especially with angiotensin-converting enzyme inhibitors (ACEI) and angiotensin II receptor blockers (ARB), which has currently been analyzed whether or not their use may contribute to a higher risk of infection and/or severity of covid-19^16^.

The covid-19 infection has a lethality of 2.2% worldwide, and currently more than four billion people have died in the world. In Brazil alone, by July 2021, more than 544,000 people died as a result of covid-19^17^. As for the relationship between hypertension and covid-19, hypertensive subjects with SARS-CoV-2 infection had 2.27 and 3.48 times higher risks of severity and fatality, respectively, compared to covid-19 cases without hypertension^18^.

In this context, the relationship between hypertension and unfavorable outcomes of covid-19 are of great concern for public health, making it essential to conduct studies that clarify the relationship between the diagnosis of hypertension and the development of severe forms of covid-19. Thus, this article aims to identify whether SAH is a risk factor for the worsening of covid-19.

## METHODS

This is a scoping review, and followed the six methodological steps described by the Joanna Briggs Institute: (1) identification of the research question; (2) identification of relevant studies; (3) selection of studies; (4) data extraction; (5) sorting, summarizing, and reporting results; and (6) dissemination of results^19^.

In order to construct the guiding question, we applied the PCC strategy, which represents a mnemonic for P (Population) (people diagnosed with SAH), C (Concept) (hospitalized people affected by covid-19), and C (Context) (covid-19 pandemic). The guiding question was defined as: Might people diagnosed with SAH have an increased risk for severe case of covid-19?

Articles was searched on the Lilacs, PubMed, SciELO, and Web of Science databases from March to April 2021 by two researchers individually, and disagreements were discussed with a third researcher, and resolved by consensus. The search terms used were: hypertension, arterial hypertension, coronavirus, covid-19, Sars-coronavirus, and Sars-CoV-2 (Box 1).

**Box 1:** Search strategies used on the databases. São Carlos, SP, 2021.

DATABASE	SEARCH STRATEGIES
PubMed	((hypertension[Title] OR “arterial hypertension”[Title]) AND (((coronavirus[Title] OR “COVID-19”[Title]) OR “SARS-CORONAVIRUS”[Title]) OR “SARS-COV-2”[Title])) NOT pulmonary[Title]
*Web of Science*	TI=(hyperttension OR “arterial hypertension”) NOT TI=(pulmonary) AND TI=(coronavirus OR Covid-19 OR SARS-CORONAVIRUS OR SARS-COV-2)
Lilacs	(hypertension OR arterial hypertension [Title words] and coronavirus OR Covid-19 OR sars-coronavirus OR Sars-cov-2 [Title words] and not pulmonary [Title words])
SciELO	TI=(hypertension OR “arterial hypertension”) NOT TI=(pulmonary) AND TI=(coronavirus OR Covid-19 OR SARS-CORONAVIRUS OR SARS-COV-2)

Searches were performed using descriptors and their synonyms found in the *Descritor em Ciências da Saúde* (DeCS) and Medical Subject Headings (MeSH) (Box 1).

We included primary studies published in Portuguese, English, and Spanish between January and December 2020, and excluded articles whose titles and abstracts were not within the objective of the investigation, and studies that addressed other risk factors, as well as opinion articles, editorials, and reviews. The reference lists of all studies found were also checked.

To select the studies, after implementing the search strategy in each database, the identified references were exported to the reference manager Mendeley, version X7.

After selecting the studies, the references were exported to the StArt (State of the Art through Systematic Review) web application for the 2-level selection of studies. The first selection was by reading titles and abstracts, followed by reading the full article. The *Laboratório de Pesquisa em Engenharia de Software* (Software Engineering Research Laboratory, LaPES) of the *Universidade Federal de São Carlos* (UFSCar).

After data mapping, PRISMA-ScR (extension for scoping reviews) was used for data extraction^20^.

## RESULTS

A total of 264 articles were identified in the databases, of which 115 were duplicates and were excluded, as well as other 127 discarded after reading the titles and abstracts, and nine after reading the full text. Therefore, 13 articles approaching the relation of systemic arterial hypertension as a risk factor for covid-19 aggravation were selected for the study (Figure).

**Figure:**
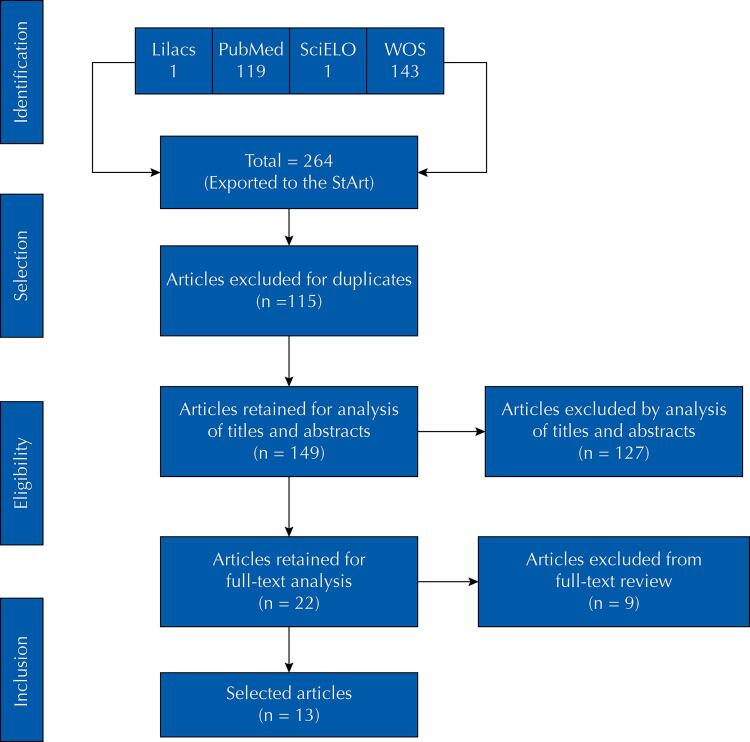
Reference flowchart: inclusion and exclusion of articles. São Carlos, SP, 2021.

The 13 publications included (100%) in this scoping review were published in English. Regarding the countries participating in the studies, the following stand out: 10 (76.9%) publications conducted in China, one in Spain (7.7%), one in Germany (7.7%), one in Turkey (7.7%); six of the 13 studies were published in journals from the United States (46.1%), four in English publications (30.8%), one in Switzerland (7.7%), one in China (7.7%), and in one Turkish publication (7.7%). Studies were published from January to December 2020, of which nine (69.2%) were retrospective observational studies and four (30.8%) were cohort studies. After selection, studies were described as to the type of study and objective, and the main results found were extracted, as shown in Box 2.

**Box 2:** Description of articles according to author, year, site, objective, type of study, sampling, and main outcomes. São Carlos, SP, 2021.

Author, Year and Site	Objective	Type of study and sampling (n)	Main results
Chen R, 2020, China^15^	Investigate the association between in-hospital blood pressure control and covid-19-related outcomes, and compare the effects of different antihypertensive treatments.	Retrospective cohort study. n = 2,828, 51.0% male, and mean age 60.0 years.	Elevated cardiomyocyte damage biomarkers in grade 2 and grade 3 groups (p < 0.001). Subjects grade 3 group higher B-type natriuretic peptide and worse cardiac function (p < 0.001). Survival rate of adverse clinical outcomes significantly higher in subjects previously treated with renin-angiotensin-aldosterone system inhibitors (HR: 0.35, 95%CI: 0.13–0.97, p = 0.043) or after (HR 0.18, 95%CI: 0.04–0.86, p = 0.031) admission than treated with other antihypertensive medications.
Rodilla E, 2020, Spain^32^	Analyze whether hypertension represents an independent risk factor for death as a difficult outcome in hospitalized subjects with SARS-CoV-2 in Spain.	Cross-sectional, observational, multicenter retrospective study. n = 12,226, mean age 67.5, and 42.6% female.	After adjustment for sex, age tertiles, and Charlson Comorbidity Index scores, hypertension was significantly predictive of all-cause mortality when treated with angiotensin-converting enzyme inhibitors (OR: 1.6, p = 0.002) or other than renin-angiotensin-aldosterone blockers (OR: 1.3, p = 0.001) or angiotensin II receptor blockers (OR: 1.2, p = 0.035).
Huang S. 2020, China^21^	Explore the effect of hypertension on disease progression and prognosis in subjects with coronavirus 2019 disease (covid-19).	Multicenter retrospective observational study. n = 310, 56.1% male, and mean age 62 years.	Comparison hypertensive and non-hypertensive subjects with covid-19 without other comorbidities: hypertension showed no significant correlation with length of stay (p = 0.409) or mortality (p = 0.189) of covid-19 disease, hypertensive subjects higher proportion of severe cases (p < 0.001), higher proportion of intensive care unit admission (p = 0.045). Comparison of laboratory indices between hypertensive subjects with covid-19 with and without other comorbidities, most laboratory indices were not significantly different.
Okay G, 2020, Turkey^22^	Investigate the effect of hypertension on clinical severity and prognosis of Coronavirus subjects with covid-19.	Retrospective observational study. n = 260, 55.4% male and mean age 54.1 ± 15.5 years.	Subjects with severe and critical clinic higher in the hypertensive than in the non-hypertensive group (p < 0.001). Greater use of oxygen therapy in hypertensive than in non-hypertensive subjects (p = 0.001). Higher rate of admission to intensive care unit in hypertensive subjects than in the non-hypertensive group (p = 0.01). Median values of neutrophil count, aspartate aminotransferase, lactate dehydrogenase and creatinine higher in hypertensive subjects than in non-hypertensive subjects. (p = 0.001; p = 0.016; p = 0.002; p < 0.001, respectively). Median albumin values and glomerular filtration rate lower in hypertensive subjects (p = 0.002 and p < 0.001, respectively).
Ghao G, 2020, China^29^	Investigate whether treatment of hypertension, primarily with renin-angiotensin-aldosterone system (RASA) inhibitors, can impact mortality in subjects with covid-19.	Retrospective observational study. n = 2,877, hypertensives n = 850 (83.5% taking antihypertensive medications).	Hypertensive subjects without antihypertensive treatment: higher mortality rate compared to those with antihypertensive treatments (7.9% vs. 3.2%, HR: 2.52, 95%CI: 1.23–5.17, p = 0.012). After adjustment, even higher mortality risk in subjects without antihypertensive treatment (HR: 2.17, 95%CI: 1.03– 4.57,= 0.041). The numerical difference in mortality rates between the renin-angiotensin-aldosterone system inhibitor and non-inhibitor cohorts were not significant before or after adjustment (2.2% vs. 3.6%, adjusted HR: 0.85, 95%CI: 0.28–2.58, p = 0.774).
Yao Q, 2020, China^24^	Explore the characteristics and differences in outcomes between hypertensive and non-hypertensive subjects with covid-19.	Observational retrospective study. n = 414, median age 61 years 50.1% male, and 36.0% hypertensive participants.	Compared with normotensives, hypertensive participants had a higher risk of death (HR: 2.68, 95%CI:1.46– 4.91), after adjustment for age and sex, no difference was shown (HR:1.77, 95%CI: 0.93–3.36). Hypertensive subjects had more complications: shock (p = 0.009), acute respiratory distress syndrome (p = 0.003), acute kidney injury (p = 0.001), greater use of non-invasive mechanical ventilation (p = 0.026), and invasive mechanical ventilation IMV (p = 0.020). Lab results on admission: hypertensive subjects had higher levels of hemoglobin (p = 0.049), D-dimer (p = 0.007), blood urea nitrogen (p = 0.000) and serum creatinine (p = 0.000).
Xiong TY, 2020, China^25^	Characterize the prevalence and clinical implications of comorbidities in subjects with covid-19.	Retrospective multicenter study. n = 472 53.0% male, median age 43 years, hypertensive subjects n = 71.	Comparison hypertensive and non-hypertensive subjects: the hypertensive ones more prone to treatments with angiotensin-converting enzyme inhibitor (ACEI)/angiotensin II receptor blocker (ARB), β-blockers, calcium channel blocker (CCB) (p < 0, 001) and statins (p = 0.006), greater chance of experiencing the composite outcome (p < 0.001) and individual outcome, including intensive care unit admission (p < 0.001), mechanical ventilation (p < 0.001) and death (p = 0.012). Occurrence of adverse events did not differ between subjects treated with and without antihypertensive drugs.
Li J, 2020, China^30^	Investigate the association between angiotensin-converting enzyme inhibitors (ACEIs) and angiotensin receptor blockers (ARBs), and disease severity and mortality in subjects with hypertension hospitalized for covid-19 infection.	Retrospective study. n = 1,178, hypertensive subjects n = 362 (52.2% male, 71.5% older than 60 years, and 31.8% were on ACEI / ARBs).	Analysis in the hypertensive group: similar laboratory profile results, except higher alkaline phosphatase in those not taking angiotensin-converting enzyme inhibitors (ACEI) / angiotensin receptor blockers (ARB) (p < 0.001), frequency of disease severity, acute respiratory distress syndrome and mortality did not differ in relation to ACEI / ARB therapy. With regard to IECA/ARB use, there was no difference between those with severe versus non-severe disease in the use of IECA (p = 0.80), ARBs (p = 0.40), or the composite of IECA / ARBs (p = 0.65). Similarly, there were no differences between non-survivors and survivors in the use of IECA (p = 0.85), ARBs (p = 0.42), or the composite of IECA / ARBs (p = 0.34).
Zhou X, 2020, China^27^	Explore the clinical features of covid-19 complicated by hypertension.	Single center retrospective study. n = 110, mean age 57.7 years, 54.5% male, hypertensive subjects n = 36 (52.8% male).	Compared to non-hypertensive subjects, those hypertensive had higher occurrence of dyspnea (p < 0.001), diabetes (p < 0.001) and cardiovascular disease (p = 0.022), lower lymphocyte count on admission (p < 0.01), higher crude mortality rate (p < 0.01). Taking angiotensin-converting enzyme inhibitors or angiotensin receptor blockers was not significantly associated with prognosis (p = 0.162).
Chengyi HU, 2020, China^26^	Determine the impact of hypertension on outcomes in subjects with covid-19.	Observational retrospective cohort study. n = 442, hypertensive subjects n = 61.	Compared to normotensive subjects, those hypertensive were more likely to develop bacterial infections (p = 0.002), higher neutrophil counts (p = 0.007), neutrophil to lymphocyte ratio (p = 0.045) and lactate dehydrogenase (p = 0.035). A higher proportion of subjects had bilateral irregular opacities on chest CT scan (p = 0.012) in the hypertension group than in the normotensive group. Hypertensive subjects were more likely to receive antibiotics (p = 0.035) and corticosteroid therapy (p = 0.035).
Pan W, 2020, China^31^	Clarify the impact of hypertension on covid-19, and investigate whether prior use of renin-angiotensin-aldosterone system (RAAS) inhibitors affects the prognosis of covid-19.	Single center retrospective study. n = 996, hypertensive subjects n = 282.	Hypertension unpaired cohort (HR 1.80, 95%CI: 1.20–2.70); paired cohort (HR 2.24, 95%CI: 1.36–3.70) independently associated with all causes of mortality in subjects with covid-19. There were no significant differences in baseline clinical characteristics between subjects with hypertension who used and did not use renin-angiotensin-aldosterone system (RASA) inhibitors. All-cause mortality rate was significantly lower in the SRAA inhibitor treatment group than in the no-SRAA inhibitor treatment group (p = 0.037).
Trump S, 2020, Germany^23^	Evaluate the effect of coexisting cardiovascular disease, in particular hypertension and antihypertensive treatment, on covid-19 pathology and viral clearance.	Prospective observational cohort study. n = 144, 67.4% male. Hypertensive subjects with or without cardiovascular disease n = 90, and subjects without hypertension and without cardiovascular disease n = 54.	Higher risk of developing critical covid-19 for hypertensive subjects with/without coexisting cardiovascular disease compared to non-hypertensive subjects (adjusted odds ratio (adjOR) = 4.28, 95%CI: confidence interval: 1.60–11.46, p = 0.028). Patients treated with angiotensin receptor blockers (ARB) increased risk of critical covid-19 compared with non-hypertensive subjects (adjOR = 4.14, 95%CI: 1.01–17.04, p = 0.044). Risk of critical disease lower than for hypertensive subjects without treatment with angiotensin-converting enzyme inhibitors (ACEIs) or ARBs (adjOR = 8.17, 95%CI: 1.65–40.52, p = 0.009). No difference in ACE2 expression and initial viral concentration between subject groups.
Yang Q, 2020, China^28^	Explore the impact of hypertension on outcomes in subjects with covid-19.	Retrospective cohort study. n = 226, hypertensive subjects n = 84.	Subjects divided into survivor and non-survivor groups. Ratio of hypertensive subjects among non-survivors was higher than among survivors (26.70% vs. 74.00%; p < 0.001). Hypertensive subjects had higher risk of death (HR: 2.679, 95%CI: 1.237–5.805; p = 0.012), elevated D-dimer levels (HR: 1.025, 95%CI: 1.011–1.039; p < 0.001) and higher neutrophil to lymphocyte ratio (HR: 1.107, 95%CI: 1.053–1.164; p < 0.001).

Of the 13 studies reviewed, three studies showed that among the subjects who developed the severe form of covid-19, the highest proportion corresponded to the group of hypertensive subjects^15,21^.

With regard to clinical complications, four studies suggested that hypertensive subjects reported higher rate of admission to the Intensive Care Unit (ICU), with clinical conditions such as shock, acute respiratory distress syndrome and acute kidney injury, in addition to greater use of invasive and non-invasive mechanical ventilation, and were more likely to receive antibiotics and corticosteroid therapy^21,22,24^.

The analysis of hypertensive subjects with and without other comorbidities found one study showing that laboratory indexes of both groups were similar^21^. On the other hand, five studies found that hypertensive subjects had higher levels of hemoglobin, neutrophils, D-dimer, aspartate aminotransferase (AST), lactate dehydrogenase (LDH) and creatinine when compared to normotensive subjects. The studies, however, also pointed out that hypertensive subjects had lower median values for albumin and glomerular filtration rate^22,24,26^.

As for mortality, one study showed that hypertensive subjects had higher risk of death when compared to normotensive subjects; however, after adjusting for age and sex, no difference was observed^24^. Two studies indicated that the mortality rate was higher in hypertensive subjects without any antihypertensive treatment, and three studies showed that the use of ACEI/ABB is not significantly associated with prognosis^25,27,29^. One study showed that after adjustment, hypertension was significantly predictive of all-cause mortality when treated with angiotensin-converting enzyme inhibitors or other than renin-angiotensin-aldosterone blockers or angiotensin II receptor blockers^32^.

## DISCUSSION

The studies presented systemic arterial hypertension as the most common chronic disease in subjects diagnosed with covid-19^15,22,31^. Most studies showed that hypertensive subjects diagnosed with covid-19 had a mean age of more than 60 years^21^.

Due to the high global prevalence of hypertension, this group of people was expected to have a high incidence of covid-19. Added to this condition, age also emerges as one of the risk factors for the development of hypertension, since during the physiological process of aging occur morphological changes, including progressive stiffening and loss of compliance of the major arteries that influence blood pressure levels^3^.

Moreover, studies prior to covid-19 have already addressed the relation of aging as an important risk factor in SARS-CoV-associated disease. One study involving young and old monkeys, both infected with the coronavirus, showed similar levels of viral replication and host response to infection. Old monkeys, however, showed stronger response to infection with increased expression of genes associated with inflammation and, according to the comparative analysis between young and old monkeys, this increase was attributable to aging^33^.

In this scenario, the hypothesis raised would be that the oxidative damage accumulated due to the aging process, added with a weakened antioxidant defense system could cause a disturbance in the balancing redox, which would cause an increase in reactive oxygen species. Thus, oxidative stress may enhance cellular responses of early mediators of inflammation. Besides affecting the innate and adaptive immune system, aging is also associated with a pro-inflammatory state in the host^33,34^.

In this scoping review, the studies showed that male subjects with systemic arterial hypertension and covid-19 were more likely to progress to severe forms of covid-19, as well as more likely to die than female subjects^28,30^. One hypothesis that could explain these findings is attributed to a potential protection of the X chromosome and sex hormones, which play an important role in the innate and adaptive development of immunity. Since the ACE2 gene is located at the Xp22 locus on the human X chromosome, the presence of alleles would confer resistance to SARS-CoV-2. It is a suggested mechanism to explain the apparent lower female susceptibility to severe covid-19 viral infection^35^.

As for the manifestation of covid-19 symptoms, some studies have shown that subjects with systemic arterial hypertension had more marked cough and dyspnea compared to the non-hypertensive group^21,22^. On the other hand, other studies have indicated that the symptoms did not differ significantly in hypertensive subjects and non-hypertensive subjects^26,29^.

It is noteworthy that the presence of cough and dyspnea are among the main manifestations observed in the moderate and severe types of covid-19^38^. In order to explain the presence or absence of symptoms found in the studies, however, one should consider each individual’s immune response to a viral infection, and analyze the variables that may influence the manifestation of symptoms in hypertensive subjects, such as smoking and history of adjacent respiratory comorbidities.

As far as drug treatment is concerned, there was no association between the use of any class of antihypertensive and a higher risk of mortality^24,31^. Subjects with systemic arterial hypertension and no antihypertensive treatment had higher mortality rate when compared to hypertensive subjects with antihypertensive treatment^29^. However, other studies showed that subjects with and without antihypertensive treatment had similar laboratory profile results, and showed no differences in the occurrence of adverse effects or clinical outcomes^25,27,30^.

It is noteworthy that because hypertension is a chronic disease, its control requires treatment with pharmacological and non-pharmacological measures throughout life^3^. Therefore, it is essential to investigate adherence to drug therapy, and lifestyle habits of hypertensive individuals.

Studies prior to the covid-19 pandemic pointed out that the high cost in the purchase of antihypertensive drugs, and the use of combinations of different pharmacological classes were presented as predictive factors for non-adherence to drug therapy, which can lead to deterioration in health^39,40^.

According to the Pan American Health Organization (PAHO), an estimated 1,130 million people worldwide are diagnosed with systemic arterial hypertension, and less than one in five has it under control. The main factors contributing to the high and growing prevalence of hypertension are unhealthy diets, especially excess sodium, insufficient potassium, physical inactivity, and alcohol consumption^2^.

Therefore, regarding the relation between presence of systemic arterial hypertension diagnosis and its impact on health when associated with SARS-CoV-2 infection, this scoping review showed that hypertensive subjects had more propensity to develop the severe form of covid-19 when compared to non-hypertensive subjects^21^. The need for ICU admission as well as the need for non-invasive and invasive ventilation were higher in hypertensive subjects when compared to non-hypertensive subjects^21,22,25,31^. As for the mortality rate, a significant increase was observed in the group of hypertensive subjects when compared to normotensive ones^22,24,28,30,31^.

It is noteworthy that during covid-19 infection, immune cell recruitment may be immune-mediated or in response to direct viral aggression to the endothelium, and may result in generalized endothelial dysfunction associated with apoptosis^41^. Hypertensive subjects already present pro-inflammatory state due to endothelial dysfunction related to the pathophysiology of systemic arterial hypertension. Disturbances in the immune system and chronic inflammatory stimulation resulting from hypertension may thus contribute to the progression of severe covid-19.

In this context, regarding laboratory findings in subjects with covid-19, studies have shown that CD3+ cells, CD4+ cells and CD8+ cells counts were lower in the hypertensive group when compared to normotensive subjects. Plasma levels of interleukin (IL) IL-6 and IL-10, however, were significantly higher in the group of hypertensive subjects with covid-19^31^. Among subjects affected by the covid-19, when compared to normotensive subjects, hypertensive subjects had significantly higher counts of D-dimer, neutrophils, neutrophil-lymphocyte ratio, WBC, lactate dehydrogenase (LDH), and creatinine^21,22,24^.

In order to explain data found, we highlight studies that have pointed to angiotensin-converting enzyme 2 (ACE2), a peptidase in the renin-angiotensin system (RAS), as the main entry receptor for SARS-CoV-2 in humans. Although endothelial cells in a variety of tissues express little or no ACE2, these cells may be infected by the virus and result in negative regulation. This has impacts on angiotensin II and SARS functions in various tissues, including the vasculature, lung, heart, and kidney^43^.

Due to the pro-inflammatory state of systemic arterial hypertension, excessive activation of coagulation pathways and platelets may still occur, as well as immune cells that will secrete cytokines, antimicrobial peptides and a variety of enzymes, and produce oxygen radicals and other media to exterminate the pathogens. However, excessive activation of these defense cells may cause damage to the respiratory epithelium, increasing the local inflammatory response, and decreasing lung function, thus enabling the covid-19 progression^43,44^.

The altered parameters are indicative that internal organs with high levels of ACE2 protein expression - such as lung, kidney, and heart - may be more vulnerable to invasion and injury by SARS-CoV-2. Hypertensive subjects tend to develop more severe covid-19, not only through severe inflammatory storms, but also through reduced protection against organ injury due to imbalances in the ACE system. Thus, delay in diagnosis and hospital admission may result in an increased risk of developing the severe form of covid-19 or even lead to death.

Finally, it should be noted that many chronic diseases, including systemic arterial hypertension, share mechanisms that lead to a pro-inflammatory state, and fading of the immune response. Thus, the body would not be able to effectively control the virus in the initial phase, which would lead to worsening of the disease in subjects with covid-19. However, in addition to the presence of hypertension in individuals with covid-19, other factors should be considered such as aging, adjacent comorbidities, history of drug treatment compliance, and unhealthy lifestyle habits that may influence the control of hypertension and consequently to the worsening of covid-19.

Finally, it is worth noting that more than 76% of the reviewed publications were from China, as expected; however, studies on the relation of systemic arterial hypertension as a risk factor for covid-19 are currently being conducted and published in different countries.

Limitations include the fact that only studies in Portuguese, English, and Spanish were included in this review; articles available in full text, and indexing databases were not included in this review.

Current literature pointed out that systemic arterial hypertension may present a poor prognosis in cases of covid-19 when compared to non-hypertensive subjects. Considering the foregoing, the results of this study reinforce the need for the development of strategies to improve assistance, as well as preventive and educational health actions directed to all hypertensive people, in addition to continuous monitoring of these people affected by covid-19.

## FINAL REMARKS

Hypertension is one of the main risk factors for adverse outcomes in subjects with covid-19. The results found in this study suggest that hypertension is associated with an increased risk of developing the severe form of covid-19, and increased mortality. However, studies addressing the effects on the outcomes of hypertensive subjects and SARS-CoV-2 infection on prior adherence to antihypertensive medications prior to covid-19 infection are still scarce, as well as studies that review the presence of other preexisting comorbidities in subjects with hypertension affected by covid-19, and how these associated diseases may contribute in the evolution to severe form.

Studies stratified by sex are needed to clarify the apparent greater susceptibility of males to the evolution of the severe form of SARS-CoV-2 infection. Future studies should include the analysis of individual variables that may influence the outcome of systemic arterial hypertension subjects infected by SARS-CoV-2, such as genetic factors, race/ethnicity, occupation, lifestyle habits, in addition to the evaluation of educational level and diagnosis of the socioeconomic and environmental situation.

## References

[1] Pan American Health Organization. World Hypertension Day – 17 May 2021. Washington, DC: PAHO; 2021 [cited 2021 Jun 14]. Available from: https://www.paho.org/en/events/world-hypertension-day-17-may-2021

[2] World Health Organization‎. Improving hypertension control in 3 million people: country experiences of programme development and implementation. Geneva (CH): WHO; 2020 [cited 2021 Jun 14]. Available from: https://apps.who.int/iris/handle/10665/336019

[3] Barroso WKS, Rodrigues CIS, Bortolotto LA, Mota-Gomes MA, Brandão AA, Feitosa ADM, et al. Diretrizes Brasileiras de Hipertensão Arterial – 2020. Arq Bras Cardiol. 2021 [cited 2021 Jun 16];116(3):516-658. Available from: http://departamentos.cardiol.br/sbc-dha/profissional/pdf/Diretriz-HAS-2020.pdf 10.36660/abc.20201238 PMC9949730 33909761

[4] Huang C, Wang Y, Li X, Ren L, Zhao J, Hu Y, et al. Clinical features of patients infected with 2019 novel coronavirus in Wuhan, China. Lancet. 2020;395(10223):497-506. 10.1016/S0140-6736(20)30183-5 PMC7159299 31986264

[5] Wang D, Hu B, Hu C, Zhu F, Liu X, Zhang J, et al. Clinical characteristics of 138 hospitalized patients with 2019 novel coronavirus-infected pneumonia in Wuhan, China. JAMA. 2020;323(11):1061-9. 10.1001/jama.2020.1585 PMC7042881 32031570

[6] Zhou F, Yu T, Du R, Fan G, Liu Y, Liu Z, et al. Clinical course and risk factors for mortality of adult inpatients with COVID-19 in Wuhan, China: a retrospective cohort study. Lancet. 2020;395(10229):1054-62. 10.1016/S0140-6736(20)30566-3 PMC7270627 32171076

[7] Tadic M, Saeed S, Grassi G, Taddei S, Mancia G, Cuspidi C. Hypertension and COVID-19: ongoing controversies. Front Cardiovasc Med. 2021;8:639222. 10.3389/fcvm.2021.639222 PMC7925389 33681308

[8] Xia F, Zhang M, Cui B, An W, Chen M, Yang P, et al. COVID-19 patients with hypertension are at potential risk of worsened organ injury. Sci Rep. 2021;11:3779. 10.1038/s41598-021-83295-w PMC7881102 33580165

[9] Garcia LB, Centurión OA. Medidas preventivas y manejo diagnóstico y terapéutico de la hipertensión arterial y las crisis hipertensivas. Rev Salud Publica Parag. 2020;10(2):59-66. 10.18004/rspp.2020.diciembre.59

[10] Wei ZY, Qiao R, Chen J, Huang J, Wu H, Wang WJ, et al. The influence of pre-existing hypertension on coronavirus disease 2019 patients. Epidemiol Infect. 2021;149:e4. 10.1017/S0950268820003118 PMC7804074 33397519

[11] Yugar-Toledo JC, Yugar LBT, Tácito LHB, Vilela-Martin JF. Disfunção endotelial e hipertensão arterial. Rev Bras Hipertens. 2015 [cited 2021 Jun 28];22(3):84-92. Available from: https://pesquisa.bvsalud.org/portal/resource/pt/biblio-881232

[12] Nägele MP, Haubner B, Tanner FC, Ruschitzka F, Flammer AJ. Endothelial dysfunction in COVID-19: current findings and therapeutic implications. Atherosclerosis. 2020;314:58-62. 10.1016/j.atherosclerosis.2020.10.014 PMC7554490 33161318

[13] Du Y, Zhou N, Zha W, Lv Y. Hypertension is a clinically important risk factor for critical illness and mortality in COVID-19: a meta-analysis. Nutr Metab Cardiovasc Dis. 2021;31(3):745-55. 10.1016/j.numecd.2020.12.009 PMC7831720 33549450

[14] Ran J, Song Y, Zhuang Z, Han L, Zhao S, Cao P, et al. Blood pressure control and adverse outcomes of COVID-19 infection in patients with concomitant hypertension in Wuhan, China. Hypertens Res. 2020;43(11):1267-76. 10.1038/s41440-020-00541-w PMC7450040 32855527

[15] Chen R, Yang J, Gao X, Ding X, Yang Y, Shen Y, et al. Influence of blood pressure control and application of renin-angiotensin-aldosterone system inhibitors on the outcomes in COVID-19 patients with hypertension. J Clin Hypertens. 2020;22(11):1974-83. 10.1111/jch.14038 PMC7537535 33006442

[16] Caldeira D, Alves M, Melo RG, António PS, Cunha N, Nunes-Ferreira A, et al. Angiotensin-converting enzyme inhibitors and angiotensin-receptor blockers and the risk of COVID-19 infection or severe disease: systematic review and meta-analysis. Int J Cardiol Heart Vasc. 2020;31:100627. 10.1016/j.ijcha.2020.100627 PMC7451091 32875060

[17] Fundação SEADE. Boletim Coronavírus Completo. São Paulo; 2021 [cited 2021 Jul 21]. Available from: https://www.seade.gov.br/coronavirus/#

[18] Zhang J, Wu J, Sun X, Xue H, Shao J, Cai W, et al. Association of hypertension with the severity and fatality of SARS-CoV-2 infection: a meta-analysis. Epidemiol Infect. 2020;148:e106. 10.1017/S095026882000117X PMC7270484 32460927

[19] Aromataris E, Munn Z, editors. JBI manual for evidence synthesis. Adelaide (AU): Joanna Briggs Institute; 2020 [cited 2021 Jun 12]. Available from: https://synthesismanual.jbi.global

[20] Tricco AC, Lillie E, Zarin W, O’Brien KK, Colquhoun H, Levac D, et al. PRISMA Extension for Scoping Reviews (PRISMA-ScR): checklist and explanation. Ann Intern Med. 2018;169(7):467-73. 10.7326/M18-0850 30178033

[21] Huang S, Wang J, Liu F, Liu J, Cao G, Yang C, et al. COVID-19 patients with hypertension have more severe disease: a multicenter retrospective observational study. Hypertens Res. 2020;43:824-31. 10.1038/s41440-020-0485-2 PMC7261650 32483311

[22] Okay G, Durdu B, Akkoyunlu Y, Bölükçü S, Kaçmaz AB, Sümbül B, et al. Evaluation of clinical features and prognosis in COVID-19 patients with hypertension: a single-center retrospective observational study. Bezmialem Sci. 2020;8 Suppl 2:15-21. 10.14235/bas.galenos.2020.4978

[23] Trump S, Lukassen S, Anker MS, Chua RL, Liebig J, Thürmann L, et al. Hypertension delays viral clearance and exacerbates airway hyperinflammation in patients with COVID-19. Nat Biotechnol. 2021;39:705-16. 10.1038/s41587-020-00796-1 33361824

[24] Yao Q, Ni J, Hu TT, Cai ZL, Zhao JH, Xie QW, et al. Clinical characteristics and outcomes in coronavirus disease 2019 (COVID-19) patients with and without hypertension: a retrospective study. Rev Cardiovasc Med. 2020;21(4):615-25. 10.31083/j.rcm.2020.04.113 33388007

[25] Xiong TY, Huang FY, Liu Q, Peng Y, Xu YN, Wei JF, et al. Hypertension is a risk factor for adverse outcomes in patients with coronavirus disease 2019: a cohort study. Ann Med. 2020;52(7):361-6. 10.1080/07853890.2020.1802059 PMC7877982 32716217

[26] Chengyi HU, Lushan X, Hongbo Z, Yanpei Z, Wenfeng Z, Li L, et al. [Effect of hypertension on outcomes of patients with COVID-19]. Nan Fang Yi Ke Da Xue Xue Bao. 2020;40(11):1537-42. Chinese. 10.12122/j.issn.1673-4254.2020.11.01 PMC7704389 33243750

[27] Zhou X, Zhu J, Xu T. Clinical characteristics of coronavirus disease 2019 (COVID-19) patients with hypertension on renin-angiotensin system inhibitors. Clin Exp Hypertens. 2020;42(7):656-60. 10.1080/10641963.2020.1764018 PMC7232880 32404011

[28] Yang Q, Zhou Y, Wang X, Gao S, Xiao Y, Zhang W, et al. Effect of hypertension on outcomes of adult inpatients with COVID-19 in Wuhan, China: a propensity score–matching analysis. Respir Res. 2020;21:172. 10.1186/s12931-020-01435-8 PMC7336415 32631365

[29] Gao C, Cai Y, Zhang K, Zhou L, Zhang Y, Zhang X, et al. Association of hypertension and antihypertensive treatment with COVID-19 mortality: a retrospective observational study. Eur Heart J. 2020;41(22):2058-66. 10.1093/eurheartj/ehaa433 PMC7314067 32498076

[30] Li J, Wang X, Chen J, Zhang H, Deng A. Association of renin-angiotensin system inhibitors with severity or risk of death in patients with hypertension hospitalized for coronavirus disease 2019 (COVID-19) infection in Wuhan, China. JAMA Cardiol. 2020;5(7):825-30. 10.1001/jamacardio.2020.1624 PMC7180726 32324209

[31] Pan W, Zhang J, Wang M, Ye J, Xu Y, Shen B, et al. Clinical features of COVID-19 in patients with essential hypertension and the impacts of renin-angiotensin-aldosterone system inhibitors on the prognosis of COVID-19 patients. Hypertension. 2020;76(3):732-41. 10.1161/HYPERTENSIONAHA.120.15289 32654555

[32] Rodilla E, Saura A, Jiménez I, Mendizábal A, Pineda-Cantero A, Lorenzo-Hernández E, et al. Association of hypertension with all-cause mortality among hospitalized patients with COVID-19. J Clin Med. 2020;9(10):3136. 10.3390/jcm9103136 PMC7650567 32998337

[33] Smits SL, Lang A, Brand JMA, Leijten LM, IJcken WF, Eijkemans MJC, et al. Exacerbated innate host response to SARS-CoV in aged non-human primates. PLoS Pathog. 2010;6(2):e1000756. 10.1371/journal.ppat.1000756 PMC2816697 20140198

[34] Weiskopf D, Weinberger B, Grubeck-Loebenstein B. The aging of the immune system. Transpl Int. 2009;22(11):1041-50. 10.1111/j.1432-2277.2009.00927.x 19624493

[35] Devaux CA, Rolain JM, Raoult D. ACE2 receptor polymorphism: susceptibility to SARS-CoV-2, hypertension, multiorgan failure, and COVID-19 disease outcome. J Microbiol Immunol Infect. 2020;53(3):425-35. 10.1016/j.jmii.2020.04.015 PMC7201239 32414646

[36] Bezman NA, Kim CC, Sun JC, Min-Oo G, Hendricks DW, Kamimura Y, et al. Molecular definition of the identity and activation of natural killer cells. Nat Immunol. 2012;13(10):1000-9. 10.1038/ni.2395 PMC3572860 22902830

[37] Jaillon S, Berthenet K, Garlanda C. Sexual dimorphism in innate immunity. Clinic Rev Allerg Immunol. 2019;56(3):308-21. 10.1007/s12016-017-8648-x 28963611

[38] Diagnosis and Treatment Protocol for Novel Coronavirus Pneumonia (Trial Version 7). Chin Med J (Engl). 2020;133(9):1087-95. 10.1097/CM9.0000000000000819 PMC7213636 32358325

[39] Mion Junior D, Silva GV, Ortega KC, Nobre F. A importância da medicação anti-hipertensiva na adesão ao tratamento. Rev Bras Hipertens. 2006 [cited 2021 Jun 27];13(1):55-8. Available from: http://departamentos.cardiol.br/dha/revista/13-1/13-importancia-da-medicacao.pdf

[40] Santana BS, Rodrigues BS, Stival MM, Volpe CRG. Arterial hypertension in the elderly accompanied in primary care: profile and associated factors. Esc Anna Nery. 2019;23(2):e20180322. 10.1590/2177-9465-EAN-2018-0322

[41] Brandão SCS, Godoi ETAM, Ramos JOX, Melo LMMP, Sarinho ESC. Severe COVID-19: understanding the role of immunity, endothelium, and coagulation in clinical practice. J Vasc Bras. 2020;19:e20200131. 10.1590/1677-5449.200131 PMC8218014 34211530

[42] Zheng Z, Peng F, Xu B, Zhao J, Liu H, Peng J, et al. Risk factors of critical & mortal COVID-19 cases: a systematic literature review and meta-analysis. J Infect. 2020;81(2):e16-25. 10.1016/j.jinf.2020.04.021 PMC7177098 32335169

[43] Barnes BJ, Adrover JM, Baxter-Stoltzfus A, Borczuk A, Cools-Lartigue J, Crawford JM, et al. Targeting potential drivers of COVID-19: neutrophil extracellular traps. J Exp Med. 2020;217(6):e20200652. 10.1084/jem.20200652 PMC7161085 32302401

[44] Newton AH, Cardani A, Braciale TJ. The host immune response in respiratory virus infection: balancing virus clearance and immunopathology. Semin Immunopathol. 2016;38(4):471-82. 10.1007/s00281-016-0558-0 PMC4896975 26965109

