# Supplementary material for: Systemic arterial hypertension as a risk factor for the severe form of covid-19: scoping review
Source: Rev Saude Publica. 2022 Mar 29;56:20. doi: 10.11606/s1518-8787.2022056004311 (PMC8973022; doi:10.11606/s1518-8787.2022056004311)
Supplement: Translation

# Translation: Hipertensão arterial sistêmica como fator de risco para a forma grave da covid-19: revisão de escopo

**Authors:** Ana Cristina Ribeiro, Sílvia Carla da Silva André Uehara

**Affiliations:** I Brasil Universidade Federal de São Carlos. Departamento de Enfermagem. São Carlos, SP, Brasil

**Keywords:** COVID-19, complicações, Fatores de Risco, Hipertensão, Revisão

## Abstract

**OBJETIVO:**

Identificar se a hipertensão arterial sistêmica se apresenta como um fator de risco para o agravamento da covid-19.

**MÉTODOS:**

Trata-se de uma revisão de escopo, as buscas foram realizadas nas bases de dados Lilacs, PubMed, SciELO e *Web of Science*.

**RESULTADOS:**

Foram selecionados 13 artigos. Os estudos apresentaram a hipertensão arterial sistêmica como a doença crônica mais comum em pacientes diagnosticados com covid-19. Os pacientes hipertensos eram mais velhos e os homens se apresentaram mais suscetíveis a desenvolver covid-19 grave. Pacientes hipertensos sem tratamento anti-hipertensivo foram associados a um maior risco de mortalidade.

**CONCLUSÕES:**

Pacientes com doenças crônicas tendem a apresentar um perfil clínico diferente, sendo essencial controlar a pressão arterial em pacientes hipertensos e monitorá-los continuamente durante a infecção por covid-19.

## INTRODUÇÃO

A hipertensão arterial sistêmica (HAS) afeta mais de 30% da população adulta, ou seja, mais de um bilhão de pessoas e se apresenta como a principal causa de morte prematura em todo o mundo^1,2^. Além disso, a carga da doença é sentida desproporcionalmente em países de baixa e média renda, onde estão dois terços dos casos, em grande parte devido ao aumento de fatores de risco nessas populações nas últimas décadas. Aproximadamente metade das pessoas que vivem com hipertensão arterial desconhecem sua condição, o que as coloca em risco de complicações médicas evitáveis e morte^2^.

Considera-se a hipertensão arterial sistêmica uma doença crônica não transmissível (DCNT), sendo uma condição multifatorial, que depende de fatores genéticos e epigenéticos, ambientais e sociais e seu ponto de corte é definido por pressão arterial sistólica (PAS) ≥ 140 e pressão arterial diastólica (PAD) ≥ 90 mm Hg^3^. Dentre os fatores de riscos para o desenvolvimento da HAS, de acordo com as Diretrizes Brasileiras de Hipertensão Arterial – 2020, destacam-se a genética, idade avançada, sexo, etnia, sobrepeso e/ou obesidade, ingestão elevada de sódio, sedentarismo, ingestão de álcool, além de fatores socioeconômicos, incluindo menor escolaridade, condições de habitação inadequadas e baixa renda familiar^3^.

Recentemente, estudos apontaram que a existência de comorbidades, como a hipertensão arterial sistêmica, em pacientes com covid-19 poderiam apresentar desfechos desfavoráveis, incluindo um risco aumentado de morte^4^. Nesse cenário, estudos identificaram que a hipertensão é a doença crônica mais comum em pacientes contaminados pelos novo coronavírus, podendo ser justificado pela elevada prevalência global^7^.

Os pacientes com hipertensão arterial sistêmica apresentam disfunção endotelial, que se apresenta como um desequilíbrio entre substâncias vasodilatadoras e vasoconstrictoras que afeta diretamente a função vascular, considerada uma importante característica do leito vascular em hipertensos. Associada ao processo de envelhecimento, a hipertensão resulta no enrijecimento progressivo e na perda de complacência das grandes artérias, sendo crucial na patogênese das complicações cardiovasculares na covid-19^11,12^.

Ressalta-se que devido ao fato da HAS se apresentar, frequentemente, assintomática, o paciente pode evoluir para alterações estruturais e/ou funcionais em órgãos-alvo, como coração, cérebro, rins e vasos, o que aumentaria a suscetibilidade para o SARS-CoV-2, elevando o risco de desfechos desfavoráveis em pessoas com covid-19^3,13^.

Os dados sobre o impacto da hipertensão arterial sistêmica conhecida e da diagnosticada recentemente em pacientes com covid-19 ainda são muito limitados. No entanto, um estudo mostrou que o controle insuficiente da pressão arterial (PA) foi independentemente associado a resultados adversos em pacientes com covid-19 e hipertensos^14^.

Nessa mesma linha, um outro estudo relatou que a hipertensão arterial sistêmica em estágio I estava presente em 37% dos pacientes hospitalizados por covid-19, enquanto a prevalência de HAS em estágios II e III era significativamente maior (61% e 70%, respectivamente). Além disso, desfechos desfavoráveis (mortalidade, choque séptico, insuficiência respiratória, síndrome do desconforto respiratório agudo (SDRA) e admissão na unidade de terapia intensiva) aumentaram gradualmente com a elevação da pressão arterial^15^.

Destaca-se ainda que a maioria dos pacientes hipertensos necessita de tratamento farmacológico, principalmente com os inibidores da enzima conversora de angiotensina (IECA) e bloqueadores dos receptores da angiotensina II (BRA) que atualmente tem sido analisado se o uso pode contribuir ou não para um maior risco de infecção e/ou gravidade da covid-19^16^.

A infecção por covid-19 possui letalidade de 2,2% no mundo e atualmente, mais de quatro bilhões de pessoas morreram no mundo, sendo que, somente no Brasil, até julho de 2021, mais de 544 mil pessoas morreram em decorrência do covid-19^17^. Quanto à relação entre hipertensão arterial sistêmica e covid-19, pacientes hipertensos com infecção por SARS-CoV-2 apresentaram riscos 2,27 e 3,48 vezes maiores de gravidade e fatalidade, respectivamente, em comparação com os casos de covid-19 sem hipertensão^18^.

Nesse contexto, a relação entre a hipertensão arterial sistêmica e desfechos desfavoráveis da covid-19 são de grande preocupação para a saúde pública, tornando-se essencial a realização de estudos que esclareçam a relação entre o diagnóstico de HAS e o desenvolvimento das formas graves da covid-19. Dessa forma, este artigo tem como objetivo identificar se a HAS se apresenta como um fator de risco para o agravamento da covid-19.

## MÉTODOS

Trata-se de uma revisão de escopo, sendo seguidos os seis passos metodológicos descritos pelo Joanna Briggs Institute: (1) identificação da questão de pesquisa; (2) identificação de estudos relevantes; (3) seleção dos estudos; (4) extração de dados; (5) separação, sumarização e relatório de resultados; e (6) divulgação dos resultados^19^.

Para a construção da questão norteadora, aplicou-se a estratégia PCC, que representa uma mnemônica para P (População) (pessoas com diagnóstico de HAS), C (Conceito) (pessoas internadas acometidas pela covid-19) e C (Contexto) (Pandemia covid-19); definida como: Pessoas com diagnóstico de HAS podem apresentar um aumento de risco para caso grave de covid-19?

A busca dos artigos foi realizada nas bases de dados Lilacs, PubMed, SciELO e *Web of Science*, no período de março a abril de 2021, por dois pesquisadores, individualmente, e as divergências foram discutidas com um terceiro pesquisador e solucionadas por consenso. Foram utilizados como termos de busca: hipertensão, hipertensão arterial, coronavírus, covid-19, Sars-coronavirus e Sars-CoV-2 (Quadro 1).

**Quadro 1:** Estratégias de busca utilizadas nas bases de dados. São Carlos, SP, 2021.

BASE DE DADOS	ESTRATÉGIAS DE BUSCA
PubMed	((hypertension[Title] OR “arterial hypertension”[Title]) AND (((coronavirus[Title] OR “COVID-19”[Title]) OR “SARS-CORONAVIRUS”[Title]) OR “SARS-COV-2”[Title])) NOT pulmonary[Title]
*Web of Science*	TI=(hyperttension OR “arterial hypertension”) NOT TI=(pulmonary) AND TI=( coronavirus OR Covid-19 OR SARS-CORONAVIRUS OR SARS-COV-2)
Lilacs	(hypertension OR arterial hypertension [Title words] and coronavirus OR covid-19 OR sars-coronavirus OR Sars-cov-2 [Title words] and not pulmonary [Title words])
SciELO	TI=(hypertension OR “arterial hypertension”) NOT TI=(pulmonary) AND TI=(coronavirus OR Covid-19 OR SARS-CORONAVIRUS OR SARS-COV-2)

As buscas foram conduzidas por meio de descritores e seus sinônimos que constam no descritor em ciências da saúde (DeCS) e no *medical subject headings* (MeSH) (Quadro 1).

Foram incluídos estudos primários publicados em português, inglês e espanhol entre janeiro e dezembro de 2020 e excluídos artigos cujos títulos e resumos não se delimitavam dentro do objetivo da investigação e estudos que abordavam outros fatores de risco, além de artigos de opinião, editoriais e revisões. Também, foram verificadas as listas de referências de todos os estudos encontrados.

Para a seleção dos estudos, após implementação da estratégia de busca em cada base de dados, as referências identificadas foram exportadas para o gerenciador de referências Mendeley, versão X7.

Após a seleção dos estudos, as referências foram exportadas para o aplicativo *web StArt (State of the Art through Systematic Review)*, para a seleção dos estudos em dois níveis. A primeira seleção foi realizada por meio da leitura de títulos e resumos, seguida pela leitura do artigo na íntegra. A ferramenta de revisão StArt foi desenvolvida pelo Laboratório de Pesquisa em Engenharia de Software (LaPES) da Universidade Federal de São Carlos (UFSCar).

Após o mapeamento dos dados, foi utilizado o PRISMA-ScR *(extension for scoping reviews)* para a extração dos dados^20^.

## RESULTADOS

Foram identificados 264 artigos nas bases de dados, dos quais 115 eram duplicatas e foram excluídos, assim como outros 127, descartados após a leitura dos títulos e resumos e nove após a leitura na íntegra. Assim, foram selecionados para o estudo 13 artigos que abordavam a relação da hipertensão arterial sistêmica como fator de risco para agravamento da covid-19 (Figura).

**Figura:** Fluxograma de referência: inclusão e exclusão dos artigos. São Carlos, SP, 2021.

As 13 publicações incluídas (100%) nesta revisão de escopo foram publicadas em inglês. Em relação aos países participantes dos estudos, destacam-se: 10 (76,9%) publicações realizadas na China, uma na Espanha (7,7%), uma na Alemanha (7,7%), uma na Turquia (7,7%); seis dos 13 estudos foram publicados em periódicos dos Estados Unidos (46,1%), quatro em publicações inglesas (30,8%), uma na Suíça (7,7%), uma na China (7,7%) e em uma publicação turca (7,7%). Os estudos foram publicados entre janeiro e dezembro de 2020, sendo nove (69,2%) estudos observacionais retrospectivos e quatro (30,8%) estudos de coorte. Após a seleção, os estudos foram descritos quanto ao tipo de estudo e objetivo, além de realizar a extração dos principais resultados encontrados, conforme apresentado no Quadro 2.

**Quadro 2:** Descrição dos artigos, segundo autor, ano, local, objetivo, tipo de estudo, amostra e principais resultados. São Carlos, SP, 2021.

Autor, ano e local	Objetivo	Tipo de estudo e amostra (n)	Principais resultados
Chen R, 2020, China^15^	Investigar a associação entre o controle da pressão arterial intra-hospitalar e os resultados relacionados à covid-19 e comparar os efeitos de diferentes tratamentos anti-hipertensivos.	Estudo de coorte retrospectivo. n = 2.828, 51,0% sexo masculino e idade média 60,0 anos.	Biomarcadores de danos aos cardiomiócitos elevados nos grupos de graus 2 e 3 (p < 0,001). Pacientes grupo grau 3 maior peptídeo natriurético do tipo B e pior função cardíaca (p < 0,001). Taxa de sobrevivência de resultados clínicos adversos significativamente maior em pacientes tratados com inibidores do sistema renina-angiotensina-aldosterona antes (HR: 0,35; lC95% 0,13–0,97; p = 0,043) ou depois (HR 0,18; IC95% 0,04–0,86; p = 0,031) da admissão do que tratadas com outros medicamentos anti-hipertensivos.
Rodilla E, 2020, Espanha^32^	Analisar se a hipertensão representa um fator de risco independente para morte como um desfecho difícil em pacientes hospitalizados com SARS-CoV-2 na Espanha.	Estudo transversal, observacional, multicêntrico retrospectivo. n = 12.226, idade média 67,5 e 42,6% sexo feminino.	Após ajuste para sexo, tercis de idade e pontuações do Índice de Comorbidade de Charlson, hipertensão foi significativamente preditiva de mortalidade por todas as causas quando tratada com inibidores da enzima de conversão da angiotensina (OR: 1,6; p = 0,002) ou outros que não bloqueadores renina-angiotensina-aldosterona (OR: 1,3; p = 0,001) ou bloqueadores do receptor de angiotensina II (OR: 1,2; p = 0,035).
Huang S. 2020, China^21^	Explorar o efeito da hipertensão na progressão da doença e prognóstico em pacientes com doença coronavírus 2019 (covid-19).	Estudo observacional retrospectivo multicêntrico. n = 310, 56,1% sexo masculino, idade média 62 anos.	Comparação hipertensos e não hipertensos com covid-19 sem outras comorbidades: hipertensão não mostrou correlação significativa com tempo de internação (p = 0,409) ou mortalidade (p = 0,189) da doença covid-19, pacientes hipertensos proporção maior de casos graves (p < 0,001), proporção maior admissão na unidade de terapia intensiva (p = 0,045). Comparação dos índices laboratoriais entre hipertensos com covid-19 com e sem outras comorbidades, maioria dos índices laboratoriais não eram significativamente diferentes.
Okay G, 2020, Turquia^22^	Investigar o efeito da hipertensão na gravidade clínica e prognóstico do coronavírus pacientes com covid-19.	Estudo observacional retrospectivo. n = 260, 55,4% sexo masculino e média de idade 54,1 ± 15,5 anos.	Pacientes com clínica grave e crítica maior nos hipertensos que no grupo não hipertenso (p < 0,001). Maior uso de terapia de oxigênio em hipertensos do que em não hipertensos (p = 0,001). Taxa de admissão para unidade de terapia intensiva em pacientes hipertensos maior do que no grupo não hipertenso (p = 0,01). Valores medianos de contagem de neutrófilos, aspartato aminotransferase, lactato desidrogenase e a creatinina maior em pacientes hipertensos do que em pacientes não hipertensos. (p = 0,001; p = 0,016; p = 0,002; p < 0,001, respectivamente). Valores medianos de albumina e taxa de filtração glomerular menores em pacientes hipertensos (p = 0,002 e p < 0,001, respectivamente).
Ghao G, 2020, China^29^	Investigar se o tratamento da hipertensão, principalmente com inibidores do sistema renina-angiotensina-aldosterona (SRAA), pode impactar a mortalidade de pacientes com covid-19.	Estudo observacional retrospectivo. n = 2.877, hipertensos n = 850 (83,5% em uso de medicamentos anti-hipertensivos).	Hipertensos sem tratamento anti-hipertensivo: taxa de mortalidade maior em comparação com aqueles com tratamentos anti-hipertensivos (7,9% vs. 3,2%; HR: 2,52; IC95% 1,23–5,17; p = 0,012). Após ajuste, risco de mortalidade ainda maior em pacientes sem tratamento anti-hipertensivo (HR: 2,17; IC95% 1,03–4,57; p = 0,041). A diferença numérica nas taxas de mortalidade entre os coortes de inibidores do sistema renina-angiotensina-aldosterona e não-inibidores não foram significativas antes ou após o ajuste (2,2% vs. 3,6%, HR ajustado: 0,85; IC95% 0,28–2,58; p = 0,774).
Yao Q, 2020, China^24^	Explorar as características e diferenças de resultados entre pacientes com e sem hipertensão com covid-19.	Estudo retrospectivo observacional. n = 414, mediana de idade 61 anos 50,1% sexo masculino e 36,0% hipertensos.	Comparação com normotensos, hipertensos apresentaram maior risco de morte (HR: 2,68; IC95% 1,46–4,91), após ajuste para idade e sexo, nenhuma diferença foi mostrada (HR: 1,77; IC95% 0,93–3,36). Hipertensos tiveram mais complicações: choque (p = 0,009), síndrome do desconforto respiratório agudo (p = 0,003), lesão renal aguda (p = 0,001), maior uso de ventilação mecânica não invasiva (p = 0,026) e ventilação mecânica invasiva VMI (p = 0,020). Resultados laboratoriais na admissão: hipertensos apresentaram maiores níveis de hemoglobina (p = 0,049) D-dímero (p = 0,007), nitrogênio da ureia no sangue (p = 0,000) e creatinina sérica (p = 0,000).
Xiong TY, 2020, China^25^	Caracterizar a prevalência e as implicações clínicas das comorbidades em pacientes com covid-19.	Estudo multicêntrico retrospectivo. n = 472 53,0% sexo masculino, mediana de idade 43 anos, hipertensos n = 71.	Comparação hipertensos e sem hipertensão: hipertensos mais propensos tratamentos com inibidor da enzima de conversão da angiotensina (IECA) / bloqueador do receptor da angiotensina II (ARB), β-bloqueadores, bloqueador dos canais de cálcio (CCB) (p < 0,001) e estatinas (p = 0,006), maior chance de experimentar o desfecho composto (p < 0,001) e desfecho individual, incluindo admissão na unidade de terapia intensiva (p < 0,001), ventilação mecânica (p < 0,001) e óbito (p = 0,012). Ocorrência de eventos adversos não diferiu entre os pacientes tratados com e sem medicamentos anti-hipertensivos.
Li J, 2020, China^30^	Investigar a associação entre inibidores da enzima de conversão da angiotensina (IECA) e bloqueadores do receptor da angiotensina (BRA) e gravidade da doença e mortalidade em pacientes com hipertensão hospitalizados por infecção por covid-19.	Estudo retrospectivo. n = 1.178, hipertensos n = 362 (52,2% sexo masculino, 71,5% mais de 60 anos e 31,8% estavam em uso de IECA / BRA).	Análise no grupo de hipertensos: resultados de perfil laboratorial semelhantes, exceto fosfatase alcalina mais alta naqueles que não tomam inibidores da enzima conversora de angiotensina (IECA) / bloqueadores do receptor da angiotensina (ARB) (p < 0,001), frequência da gravidade da doença, síndrome da angústia respiratória aguda e mortalidade não diferiu em relação à terapia IECA / ARB. Com relação ao uso de IECA / ARB, não houve diferença entre aqueles com doença grave versus não grave no uso de IECA (p = 0,80), ARBs (p = 0,40), ou o composto de IECA / ARBs (p = 0,65). Da mesma forma, não houve diferenças entre não sobreviventes e sobreviventes no uso de IECA (p = 0,85), ARBs (p = 0,42) ou o composto de IECA / ARBs (p = 0,34).
Zhou X, 2020, China^27^	Explorar as características clínicas do covid-19 complicadas pela hipertensão.	Estudo retrospectivo de centro único. n = 110, idade média 57,7 anos, 54,5% sexo masculino, hipertensos n = 36 (52,8% sexo masculino).	Comparados aos não hipertensos, hipertensos apresentaram ocorrência maior de dispneia (p < 0,001), diabetes (p < 0,001) e doença cardiovascular (p = 0,022), menor contagem de linfócitos na admissão (p < 0,01), taxa bruta de mortalidade maior (p < 0,01). Tomar inibidores da enzima conversora de angiotensina ou bloqueadores do receptor da angiotensina não foi significativamente associado ao prognóstico (p = 0,162).
Chengyi HU, 2020, China^26^	Determinar o impacto da hipertensão nos resultados de pacientes com covid-19.	Estudo observacional de Coorte retrospectivo. n = 442, hipertensos n = 61.	Comparação aos normotensos, hipertensos mais propensos a desenvolver infecções bacterianas (p = 0,002), contagens mais altas de neutrófilos (p = 0,007), relação neutrófilos / linfócitos (p = 0,045) e lactato desidrogenase (p = 0,035). Proporção maior de pacientes apresentou opacidades irregulares bilaterais na tomografia computadorizada de tórax (p = 0,012) no grupo de hipertensão do que no grupo de normotenso. Hipertensos mais propensos a receber antibióticos (p = 0,035) e terapias com corticosteroides (p = 0,035).
Pan W, 2020, China^31^	Esclarecer o impacto da hipertensão na covid-19 e investigar se o uso prévio de inibidores do sistema renina-angiotensina-aldosterona (RAAS) afeta o prognóstico do covid-19.	Estudo retrospectivo de centro único. n = 996, hipertensos n = 282.	Hipertensão coorte não pareada (HR 1,80; IC95% 1,20 –2,70); coorte pareada (HR 2,24; IC95% 1,36–3,70) independentemente associada com todas as causas de mortalidade em pacientes com covid-19. Não houve diferenças significativas nas características clínicas básicas entre os pacientes com hipertensão que usaram e não usaram inibidores do sistema renina-angiotensina-aldosterona (SRAA). Taxa de mortalidade por todas as causas foi significativamente menor no grupo de tratamento com inibidores SRAA do que no grupo de tratamento sem inibidor de SRAA (p = 0,037).
Trump S, 2020, Alemanha^23^	Avaliar o efeito de doenças cardiovasculares coexistentes, em particular de hipertensão e tratamento anti-hipertensivo, na patologia de covid-19 e na depuração viral.	Estudo de coorte observacional prospectivo. n = 144, 67,4% sexo masculino. Pacientes hipertensos com ou sem doenças cardiovasculares n = 90 e pacientes sem hipertensão e sem doenças cardiovasculares n = 54.	Maior risco de desenvolver covid-19 crítico para hipertensos com/sem doença cardiovascular coexistente em relação a pacientes não hipertensos (odds ratio ajustada (adjOR) = 4,28, intervalo de confiança de IC95% 1,60–11,46; p = 0,028). Pacientes tratados com bloqueadores do receptor da angiotensina (ARB) risco aumentado de covid-19 crítico em comparação com pacientes não hipertensos (adjOR = 4,14; IC95% 1,01–17,04; p = 0,044). Risco de doença crítica menor do que para pacientes hipertensos sem tratamento com inibidores da enzima conversora de angiotensina (IECA) ou ARB (adjOR = 8,17; IC95% 1,65–40,52; p = 0,009). Sem diferença na expressão de ACE2 e na concentração viral inicial entre os grupos de pacientes.
Yang Q, 2020, China^28^	Explorar o efeito da hipertensão nos desfechos de pacientes com covid-19.	Estudo de coorte retrospectivo. n = 226, hipertensos n = 84.	Participantes divididos em grupos de sobreviventes e não sobreviventes. Proporção de hipertensos entre os não sobreviventes foi maior do que entre os sobreviventes (26,70% vs. 74,00%; p < 0,001). Hipertensos com risco maior de morte (HR: 2,679; IC95% 1,237–5,805; p = 0,012), níveis elevados de dímero D (HR: 1,025; IC95% 1,011–1,039; p < 0,001) e razão de neutrófilos para linfócitos superiores (HR: 1,107; IC95% 1,053–1,164; p < 0,001).

Dos 13 estudos analisados, três estudos mostraram que, dentre os pacientes que desenvolveram a forma grave da covid-19, a maior proporção correspondia ao grupo de hipertensos^15,21^.

No que se refere às complicações clínicas, quatro estudos indicaram que os hipertensos tinham maior taxa de admissão em unidade de terapia intensiva, apresentando condições clínicas como choque, síndrome do desconforto respiratório agudo e lesão renal aguda, além do maior uso de ventilação mecânica invasiva e não invasiva, além de mais propensos a receber antibióticos e terapias com corticosteroides^21,22,24^.

Na análise de hipertensos com e sem outras comorbidades, um estudo evidenciou que os índices laboratoriais de ambos os grupos foram semelhantes^21^. Por outro lado, cinco estudos identificaram que pacientes hipertensos apresentaram níveis mais altos de hemoglobina, neutrófilos, D-dímero, aspartato aminotransferase (AST), lactato desidrogenase (LDH) e a creatinina quando comparados com os normotensos. Entretanto, também apontaram que pacientes hipertensos apresentaram valores medianos menores de albumina e taxa de filtração glomerular^22,24,26^.

No que se refere à mortalidade, um estudo mostrou que os hipertensos apresentaram maior risco de morte quando comparados com os normotensos; porém, após ajuste para idade e sexo, nenhuma diferença foi observada^24^. Dois estudos indicaram que a taxa de mortalidade foi maior em hipertensos sem qualquer tratamento anti-hipertensivo e três estudos evidenciaram que o uso de IECA/BRA não está significativamente associado ao prognóstico^25,27,29^. Além disso, um estudo mostrou que, após ajuste, a hipertensão foi significativamente preditiva de mortalidade por todas as causas quando tratada com inibidores da enzima de conversão da angiotensina ou outros que não bloqueadores renina-angiotensina-aldosterona ou bloqueadores do receptor de angiotensina II^32^.

## DISCUSSÃO

A hipertensão arterial sistêmica foi identificada como a doença crônica mais comum em pacientes diagnosticados com covid-19^15,22,31^. Além disso, a maioria dos estudos mostrou que os pacientes hipertensos com diagnóstico de covid-19 possuíam média de idade superior a 60 anos^21^.

Devido à elevada prevalência mundial da hipertensão arterial sistêmica, era previsível que esse grupo de pessoas apresentassem uma elevada incidência de covid-19. Agregado a essa condição, a idade também se apresenta como um dos fatores de risco para o desenvolvimento da hipertensão, uma vez que durante o processo fisiológico do envelhecimento, ocorrem alterações morfológicas e incluem o enrijecimento progressivo e perda de complacência das grandes artérias que influenciam nos níveis da pressão arterial^3^.

Ademais, estudos anteriores à covid-19 já abordavam a relação do envelhecimento como um importante fator de risco na doença associada ao SARS-CoV. Destaca-se um estudo envolvendo macacos jovens e idosos, ambos infectados com o coronavírus, em que os níveis de replicação viral e a resposta do hospedeiro à infecção eram semelhantes. Entretanto, os macacos idosos apresentaram uma resposta mais forte à infecção com aumento na expressão de genes associados à inflamação e segundo a análise comparativa entre macacos jovens e idosos, esse aumento era resultado atribuído ao envelhecimento^33^.

Nesse cenário, a hipótese levantada seria de que o dano oxidativo acumulado devido ao processo de envelhecimento e um sistema de defesa antioxidante enfraquecido poderiam causar um distúrbio no equilíbrio redox, o que causaria um aumento de espécies reativas de oxigênio. Assim, o estresse oxidativo pode potencializar as repostas celulares de mediadores precoces da inflamação e além do envelhecimento afetar o sistema imunológico inato e adaptativo, também está associado a um estado pró-inflamatório no hospedeiro^33,34^.

Nesta revisão de escopo, os estudos apresentaram que pacientes do sexo masculino com hipertensão arterial sistêmica e covid-19, apresentaram maior susceptibilidade para evoluir para as formas graves da covid-19, bem como maior probabilidade de ir a óbito, do que pacientes do sexo feminino^28,30^. Uma hipótese para explicar esses achados se atribui a uma possível proteção do cromossomo X e dos hormônios sexuais, que desempenham um papel importante no desenvolvimento inato e adaptativo da imunidade, uma vez que o gene ACE2 está localizado no locus Xp22 no cromossomo X humano, a presença de alelos conferiria resistência ao SARS-CoV-2, sendo um mecanismo sugerido para explicar a aparente menor susceptibilidade feminina à infecção viral por covid-19 grave^35^.

Quanto à manifestação dos sintomas de covid-19, alguns estudos mostraram que os pacientes com hipertensão arterial sistêmica apresentaram quadros de tosse e dispneia mais acentuados em comparação com o grupo não hipertenso^21,22^. Por outro lado, outros estudos indicaram que os sintomas não diferiram significativamente nos pacientes hipertensos e nos pacientes não hipertensos^26,29^.

Ressalta-se que a presença de tosse e dispneia estão entre as principais manifestações observadas no tipo moderado e grave da covid-19^38^. No entanto, para explicar a presença ou ausência dos sintomas encontrados nos estudos, deve-se considerar a resposta imunológica de cada indivíduo diante de uma infecção viral, além disso, é fundamental a análise de variáveis que possam influenciar quanto a manifestação dos sintomas em hipertensos, como o tabagismo e histórico de comorbidades respiratórias adjacentes.

No que se refere ao tratamento medicamentoso, o uso de qualquer classe de anti-hipertensivos não foi associado a um maior risco de mortalidade^24,31^. Pacientes com hipertensão arterial sistêmica sem tratamento anti-hipertensivo apresentaram maior taxa de mortalidade quando comparados aos pacientes hipertensos com tratamentos anti-hipertensivos^29^. Entretanto, outros estudos apresentaram que pacientes com e sem tratamento anti-hipertensivo tiveram resultados de perfil laboratorial semelhantes e não apresentaram diferenças na ocorrência de efeitos adversos ou nos resultados clínicos^25,27,30^.

Destaca-se que pelo fato de a hipertensão arterial sistêmica ser uma doença crônica, seu controle requer tratamento com medidas farmacológicas e não farmacológicas no decorrer de toda a vida^3^. Dessa forma, é essencial investigar a adesão à terapia medicamentosa e aos hábitos de vida dos indivíduos hipertensos.

Estudos anteriores à pandemia de covid-19 apontaram que o custo elevado na compra dos medicamentos anti-hipertensivos e o uso de combinações de classes farmacológicas diferentes se apresentaram como fatores preditivos para a não adesão à terapia medicamentosa, o que pode levar a deterioração na saúde^39,40^.

Segundo a Organização Pan-Americana da Saúde (OPAS), estima-se que 1.130 milhão de pessoas no mundo possuem diagnóstico de hipertensão arterial sistêmica, e menos de uma em cada cinco a tem sob controle. Os principais fatores que contribuem para a alta e crescente prevalência de hipertensão arterial são as dietas não saudáveis, especialmente o excesso de sódio, potássio insuficiente, inatividade física e consumo de bebidas alcoólicas^2^.

Sendo assim, quanto a relação da presença de diagnóstico de hipertensão arterial sistêmica e o seu impacto sobre a saúde quando associado à infecção pelo SARS-CoV-2, esta revisão de escopo mostrou que os pacientes hipertensos apresentaram uma maior propensão de desenvolver a forma grave da covid-19 quando comparados aos pacientes não hipertensos^21^. Além disso, a necessidade de admissão em unidade de terapia intensiva, assim como a necessidade de ventilação não invasiva e invasiva foram maiores nos pacientes hipertensos quando comparados aos pacientes não hipertensos^21,22,25,31^ e, quanto a taxa de mortalidade, observou-se um aumento significativo no grupo de hipertensos quando comparados aos normotensos^22,24,28,30,31^.

Destaca-se que durante a infecção pela covid-19, o recrutamento de células imunes pode ser imunomediado ou em resposta pela agressão viral direta ao endotélio, podendo resultar em disfunção endotelial generalizada associada à apoptose^41,42^. Contudo, pacientes hipertensos já apresentam um estado pró-inflamatório decorrente da disfunção endotelial relacionada à fisiopatologia da hipertensão arterial sistêmica. Dessa forma, os distúrbios no sistema imunológico e a estimulação inflamatória crônica resultante da hipertensão podem contribuir para a progressão da covid-19 grave.

Nesse contexto, no que se refere aos resultados laboratoriais de pacientes com covid-19, estudos mostraram que as contagens de células CD3+, células CD4+ e células CD8+ foram menores no grupo de hipertensos quando comparados aos pacientes normotensos. No entanto, os níveis plasmáticos de interleucina (IL) IL-6 e IL-10 foram significativamente maiores no grupo de hipertensos com covid-19^31^. Dentre os pacientes acometidos pela covid-19, quando comparados aos pacientes normotensos, os pacientes hipertensos apresentaram contagens significativamente maiores de dímero D, neutrófilos, proporção de neutrófilos-linfócitos, leucócitos, lactato desidrogenase (LDH) e creatinina^21,22,24^.

Desse modo, para explicar os dados encontrados, destaca-se estudos que têm apontado a enzima conversora de angiotensina 2 (ECA2), uma peptidase no sistema renina-angiotensina (SRA), como o principal receptor de entrada para o SARS-CoV-2 em humanos. Embora as células endoteliais em uma variedade de tecidos expressem pouco ou nenhuma ECA2, essas células podem ser infectadas pelo vírus e resultar em uma regulação negativa impactando nas funções da angiotensina II e SRA em vários tecidos, incluindo a vasculatura, pulmão, coração e rim^43^.

Além disso, devido ao estado pró-inflamatório da hipertensão arterial sistêmica, ainda pode ocorrer ativação excessiva de vias de coagulação e plaquetas, assim como de células imunes que irão secretar citocinas, peptídeos antimicrobianos e uma variedade de enzimas, além de produzir radicais de oxigênio e outros mediadores para exterminar os patógenos. Contudo, a ativação excessiva dessas células de defesa pode causar danos ao epitélio respiratório, aumentando a resposta inflamatória local e diminuindo a função pulmonar, proporcionando a progressão da covid-19^43,44^.

Diante de parâmetros alterados, que se apresentam como indicativos de que órgãos internos com altos níveis de expressão da proteína ECA2, como pulmão, rim e coração podem ser mais vulneráveis à invasão e lesão pelo SARS-CoV-2. Pacientes hipertensos tendem a desenvolver covid-19 mais grave, não apenas por meio de tempestades inflamatórias graves, mas também por meio de proteção reduzida contra lesão de órgão devido a desequilíbrios no sistema ECA. Assim, o atraso no diagnóstico e admissão hospitalar pode resultar em um risco aumentado de desenvolver a forma grave da covid-19 ou até mesmo levar a morte.

Por fim, é necessário destacar que muitas doenças crônicas, incluindo a hipertensão arterial sistêmica, partilham mecanismos que levam a um estado pró-inflamatório e atenuação da resposta imunológica. Dessa forma, o organismo não poderia controlar efetivamente o vírus na fase inicial, o que levaria ao agravamento da doença em pacientes com covid-19. No entanto, além da presença da hipertensão arterial em indivíduos com covid-19, deve-se levar em consideração outros fatores como o envelhecimento, comorbidades adjacentes, histórico de adesão ao tratamento medicamentoso e hábitos de vida não saudáveis que podem influenciar no controle da hipertensão e consequentemente para o agravamento da covid-19.

Por fim, vale ressaltar que mais de 76% das publicações revisadas foram oriundas da China, conforme esperado; entretanto, atualmente estudos sobre a relação da hipertensão arterial sistêmica como um fator de risco para o covid-19 estão sendo realizados e publicados em diferentes países.

Consideram-se como limitações o fato de terem sido incluídos nesta revisão somente estudos em português, inglês e espanhol, artigos que se encontravam disponíveis em texto na íntegra e bases de indexação não incluídos nesta pesquisa.

A literatura atual tem apontado que a hipertensão arterial sistêmica pode apresentar um mau prognóstico em casos de covid-19 quando comparados aos pacientes não hipertensos. Diante do exposto, os resultados deste estudo reforçam a necessidade de desenvolvimento de estratégias para melhorar a assistência, bem como de ações preventivas e educativas de saúde direcionadas para todas as pessoas hipertensas, além de um monitoramento contínuo com essas pessoas acometidas pela covid-19.

## CONSIDERAÇÕES FINAIS

A hipertensão arterial sistêmica se apresenta como um dos principais fatores de risco para os resultados adversos de pacientes com covid-19. Os resultados encontrados neste estudo sugerem que a hipertensão arterial está associada a um risco aumentado do desenvolvimento da forma grave da covid-19 e aumento da mortalidade. No entanto, ainda são escassos os estudos que abordem os efeitos nos resultados de pacientes hipertensos e a infecção pelo SARS-CoV-2 quanto à adesão prévia no uso de medicamentos anti-hipertensivos anterior à infecção pela covid-19, bem como analisar a presença de outras comorbidades preexistentes em pacientes com hipertensão acometidos pela covid-19 e como essas doenças associadas podem contribuir na evolução para forma grave.

Além disso, é necessária a realização de estudos estratificados por sexo para esclarecer a aparente maior suscetibilidade do sexo masculino à evolução da forma grave da infecção pelo SARS-CoV-2.É essencial em estudos futuros a inclusão da análise de variáveis individuais que possam influenciar no desfecho de pacientes com hipertensão arterial sistêmica infectados pelo SARS-CoV-2, como fatores genéticos, raça/etnia, ocupação, hábitos de vida, além da avaliação do grau educacional e diagnóstico da situação socioeconômica e ambiental.
